# Immunostaining for DNA Modifications: Computational Analysis of Confocal Images

**DOI:** 10.3791/56318

**Published:** 2017-09-07

**Authors:** Ashley H. Ramsawhook, Lara C. Lewis, Maria Eleftheriou, Abdulkadir Abakir, Paulina Durczak, Robert Markus, Seema Rajani, Nicholas R.F. Hannan, Beth Coyle, Alexey Ruzov

**Affiliations:** ^1^Division of Cancer and Stem Cells, School of Medicine, Centre for Biomolecular Sciences, University of Nottingham; ^2^School of Life Sciences Imaging (SLIM), School of Life Sciences, University of Nottingham; ^3^Children's Brain Tumour Research Centre, School of Medicine, QMC, University of Nottingham

**Keywords:** Genetics, Issue 127, Epigenetics, DNA, oxi-mCs, N6-methyladenine, Immunohistochemistry, Zen, Confocal Microscopy, Signal Quantitation, Spatial Distribution

## Abstract

For several decades, 5-methylcytosine (5mC) has been thought to be the only DNA modification with a functional significance in metazoans. The discovery of enzymatic oxidation of 5mC to 5-hydroxymethylcytosine (5hmC), 5-formylcytosine (5fC) and 5-carboxylcytosine (5caC) as well as detection of N6-methyladenine (6mA) in the DNA of multicellular organisms provided additional degrees of complexity to the epigenetic research. According to a growing body of experimental evidence, these novel DNA modifications may play specific roles in different cellular and developmental processes. Importantly, as some of these marks (e. g. 5hmC, 5fC and 5caC) exhibit tissue- and developmental stage-specific occurrence in vertebrates, immunochemistry represents an important tool allowing assessment of spatial distribution of DNA modifications in different biological contexts. Here the methods for computational analysis of DNA modifications visualized by immunostaining followed by confocal microscopy are described. Specifically, the generation of 2.5 dimension (2.5D) signal intensity plots, signal intensity profiles, quantification of staining intensity in multiple cells and determination of signal colocalization coefficients are shown. Collectively, these techniques may be operational in evaluating the levels and localization of these DNA modifications in the nucleus, contributing to elucidating their biological roles in metazoans.

**Figure:**
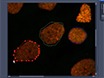


## Introduction

DNA methylation, a well-documented mechanism associated with transcriptional regulation entails modification of cytosine residues in a 5'-cytosine-phosphate-guanine-3' (CpG) dinucleotide context via the addition of a methyl group (-CH_3_) to the 5- carbon atom of the cytosine pyrimidine ring to form 5-methylcytosine (5mC)^1^. In mammals, approximately 70% of CpGs are methylated which constitutes only 1% of their genomes as they are depleted of this palindromic sequence owing to 5mC mutagenic propensity to spontaneously deaminate to thymine^2^. Presence of methyl groups on gene promoter sequences show strong correlation with transcriptional repression in vertebrates^3^,^4^,^5^. Addition of these methyl groups is catalyzed by highly conserved DNA methyltransferase (DNMT) enzymes DNMT3a, 3b and 3L, and DNMT1 which modify CpG cytosines in *de novo* and maintenance methylation contexts respectively^6^. DNMT3A/B expression is elevated during development in embryonic stem cells and epiblast; however its diminished expression is observed following pluripotent cell lineage commitment to somatic fates during differentiation^8^. Whilst sharing functional redundancy, DNMT3a and 3b display tissue-specific expression patterns, with 3a detected uniformly in mouse embryos but 3b predominantly localized to neuroectoderm and chorionic ectoderm tissues^8^.

Methylation signatures can be inherited during mitosis and meiosis^10^. Maintenance methylation involves DNMT1 facilitated modification of CpG cytosine residues existing in hemi-methylated palindromes on double-stranded DNA^11^. DNMT1 binds DNA at replication forks^11^ and consequently, genomic methylation levels peak during S phase of the cell cycle^12^. DNMT1 methylates unmodified cytosines thereby distinguishing newly synthesized DNA strands, promoting X-chromosome inactivation and maintaining transcriptional repression profiles^13^. Through recognition of hemi-methylated DNA CpG sequences, DNMT1 maintains established patterns of methylation demarcated by *de novo* methylation *e.g. *repression of Long Interspersed Nuclear Element 1 (*LINE1*) retrotransposon promoters to inhibit its potentially carcinogenic propagation^14^. Although possessing hemi-methylated DNA preferential binding affinity, DNMT1 can methylate unmethylated CpG island (CGI) sequences in *DNMT3a/b^ -/-^* mutant cells, fulfilling an emergency *de novo* methylation role^15^. Thus owing to the semi-conservative nature of DNA replication^16^, maintenance methylation can faithfully recapitulate methylation signatures from parent to daughter cell^17^.

However, for gene expression to be dynamically modulated, repressive methylation modification must be erased, which can be achieved by passive and active demethylation mechanisms^18^. The Ten-Eleven Translocase (TET) proteins belonging to a conserved family of dioxygenase proteins are capable of iterative oxidation of methyl groups on CpG residues^19^. These Tet proteins, homologous to J-Base binding proteins (JBP) discovered in *Trypanosome bruceii*, recognise and bind modified DNA bases e.g. 5mC and oxygenate these residues to 5-hydroxymethylcytosine (5hmC), 5-formylcytosine (5fC) and 5-carboxylcytosine (5caC)^20^. Tet protein facilitated oxidation of 5mC results in stepwise conformation change from methyl to hydroxyl, carbonyl and carboxylate configurations, however 5fC and 5caC modifications can be synthesized directly from 5mC oxidation^21^,^22^,^23^,^24^.

An informative indication of TET protein activity in understanding their regulation is studying the distribution and abundance of oxi-methyl-cytosine marks. Significant 5mC presence at CpG poor promoters is detectable in contrast to unmethylated CpG rich regions, the latter being characteristic of CpG islands^25^. Across tissues, highest tissue specific methylation is observed in brain, testis and blood whilst oral mucosa exhibit greatest hypomethylation, indicating a differential methylation pattern occurring at tissue specific promoters^26^.

Through utilizing sensitive anti-5mC and anti-5hmC antibodies in selective methyl/hydroxymethyl DNA immunoprecipitation (meDIP/hmeDIP), and subsequent high throughput sequencing, Ficz *et al.* demonstrated high 5hmC occupancy at promoters, exons and LINE-1 retrotransposon sequences which correlated with reduced 5mC levels at these locations in mouse embryonic stem cells^27^. Inversely, greatest 5mC enrichment was observed at repetitive satellite sequences where 5hmC presence was limited^28^. Studies performed on human frontal lobe brain tissue reveal highly significant 5hmC enrichment, four fold higher than in mouse embryonic stem cells^28^. In concordance with previous observations, high throughput sequencing of frontal lobe tissue illustrated majority 55-59% of 5hmC signal localized at low density CpG promoter regions, 35-38% within gene bodies and approximately 6% occupancy at intergenic regions. In contrast, 5mC was enriched at intergenic regions (25-26%) and higher within gene bodies (52-55%) but reduced (22-24%) at promoter sequences^29^. These studies indicate abundance of 5hmC in embryonic stem cells and somatic tissue, particularly the brain, however investigations on 5fC and 5caC distributions are limited.

Interestingly, recently discovered in eukaryotes, methylation of adenine residues at the position 6 nitrogen (N^6^) (6mA) display a genomic abundance profile inverse to that of 5mC^30^. Observations from liquid chromatography coupled tandem mass spectrometry reveal 6mA absolute levels to exist in excess in zebra fish and porcine early embryos compared to sperm with its levels (0.003% of genomic adenines) increasing steadily upon fertilization, peaking at the morula developmental stage (33 fold higher than sperm) and reaching steady state somatic levels of 0.004% of genomic adenines^31^. Immunoprecipitation of 6mA enriched DNA sequences has demonstrated predominant occupancy (approximately 80% enrichment) of this mark at repetitive element regions and transcriptional start sites^32^. These observations contextualize and validate the discovery of 6mA demethylase-null embryonic stem cells exhibiting accumulated 6mA mediated LINE-1 retrotransposon silencing compared to transcriptionally active elements in wildtype cells. These data suggest a transcriptional regulatory function for 6mA^33^.

Whilst conjugated biochemical tags coupled to subsequent DIP assays indicate presence or absence of oxidized methylcytosine derivatives (oxi-mCs), they cannot impart spatial distribution or quantifiable information of these marks^34^,^35^. A protocol for sensitive immunochemical detection of 5hmC and 5caC was recently developed^36^. This fluorophore conjugated secondary antibody-based immunostaining method coupled with utilizing scanning laser confocal microscopy possesses the unique advantage of providing visual localization of these DNA modifications within the cells, thus, emphasizing individual positively or negatively stained cells corresponding with the heterogeneous presence of these marks. The 5hmC and 5caC absolute signal intensities as amplified by the conjugated antibodies enable semi-quantitative interpretations to be proposed about the magnitude and positions of these marks within the nucleus e.g. heterochromatic and euchromatic regions^37^,^38^. Here a technique for the computational analysis of confocal microscopy images is described. The generation of 2.5D spatial distribution plots for displaying distinct 5hmC and 5caC signal peaks per pixel and their locations within nuclei is demonstrated. Histogram plots of 5hmC and 5caC signal intensity profiles can illustrate trends in abundance of these marks as the peaks and troughs are plotted as separate non-overlapping channels. Finally, by implementing the colocalization function, the degree of proximity of one signal to another can be determined and as a result of this, their respective genomic coordinates can be identified.

## Protocol

### 1. Generation of Confocal Images

1. In preparation for the analysis of modified forms of cytosine, perform immunohistochemical staining as described by Abakir *et al.*^34^.
2. Carry out imaging of slides out using a microscope and save files in an LSM format. NOTE: When comparing intensity profiles between samples the values used for laser power and gain for each channel must be maintained throughout the image taking procedure to allow for direct comparison at the image analysis stage.

### 2. Generation of 2.5D Intensity Plots

1. Open the software (*e.g.*, Zen Black) and select 'Image Processing'.
2. Go to the File menu and select Open. Select the image that requires processing.
3. **Click on 'Show All' below the image to make all graphical controls visible. In the lower half of the screen there are three tabs; 'Dimensions', 'Display' and 'Graphics', select the 'Graphics' tab.**
  1. Choose the 'rectangle' tool to select nuclei/area of interest.
  2. Select 'Cut region' to crop the image. This will generate a new image for the cropped region in a separate tab.
  3. Go to the File menu and select Save As. Give the file a name and save it as an LSM or CZI file.
4. Open software (*e.g.*, Zen Blue) and select the 'Zen Desk'.
5. Send the cropped image from Zen Black to Zen Blue by selecting the file and clicking on the File menu and select Send to Zen 2012-blue edition. The cropped image will then open in the relevant program. NOTE: The image file is transferred because the 2.5D intensity plot feature in Zen Black 2012 does not display multiple channel images simultaneously.
6. On the left hand side of the image choose the tab labelled '2.5D', this enables visualization of the image in 2.5D. Click 'Show All' to make all graphical controls visible.
7. Alter visualization settings using four grey bars situated to the left and below the image. NOTE: Left hand bar, top of the screen = zoom. Left hand bar, bottom of the screen = turn image on axis. Bottom of screen, left hand bar = rotation of image. Bottom of screen, right hand bar = expand and contract scale bars.
8. Ensure that render mode selected is surface as this gives the best visualization of the individual peaks.
9. Alter grid distance by changing the percentage, the lower the percentage the more defined each individual peak.
10. To save the 2.5D image, first hide the file list on the right side and the graphical tools below the image by clicking on the grey arrow below the 2.5D plot (next to 'show all'), this will hide the graphical tabs allowing for the image to expand to fill the screen. To save the 2.5D plot, press the 'PrtSc' key on the keyboard. This will capture a screenshot of the display. Paste the screenshot in Microsoft Paint and crop the image before saving.

### 3. Intensity Profiling

1. Open the software (*e.g.*, Zen Black) and select Image Processing.
2. Go to the File menu and select Open. Select the image that requires processing.
3. AS the image will appear on the screen, on the left hand side of the image select the tab labelled 'Profile'.
4. In the lower half of the screen select (tick) the 'Show All' tab. This will enable access to all formatting options.
5. In the lower half of the screen there are three tabs; 'Dimensions', 'Display' and 'Profile'; select 'Profile' and select (tick) the 'Table' box. Select the 'Arrow' button.
6. On the image use the mouse to select a start point and drag the mouse over a continuous number of cells. Release the mouse to produce an intensity plot for the cells along the line; additionally the table will be populated by intensity measurement data for the pixels along the line for each wavelength. NOTE: The table will provide the distance (µm) at which pixel intensity is read for the red, green and blue fluorescence.
7. To export this data, right click on the table, select 'Save table…' and save to an appropriate location as a .txt file.
8. To save the selected intensity profile image select the File menu and select 'Export…'. In the pop-up window choose 'Tagged Image File (Format)' and 'Contents of Image Window - single pane (Data)' followed by clicking 'Select file name and save…' choose an appropriate location and click 'Save'.
9. Repeat the process for each individual Intensity profile depending on the fields of vision and number of profiles to analyze.
10. Import intensity profiles data saved (see 3.7) and analyze in a spreadsheet followed by statistical analysis.

### 4. Co-localization Analysis

1. Open the software and select 'Image processing'.
2. Go to the File menu and select Open. Select the image that requires processing.
3. As the image will appear on the screen, on the left hand side of the image select the tab labelled 'Coloc'.
4. In the lower half of the screen select (tick) the 'Show All' tab. This will enable access to all formatting options.
5. **In the lower half of the screen there are four tabs; 'Dimensions', 'Display', 'Graphics' and 'Colocalization'; select 'Colocalization' and tick the box for 'Table' and 'Image'.**
  1. Select the third icon along at the top of this tab (Close Bezier text will appear when the mouse hovers over the tool) then use this tool to encircle a single nuclei, values for this nuclei will then appear in the table. NOTE: For each encircled nuclei a scatter plot is produced for red versus green fluorescence which is visualized in the left hand panel on the screen. The axis is then moved in order to gate out cells depending on the threshold. As the nuclei of interest are selected manually in the images, adjusting the gating thresholds above zero is not required. All pixel intensity values observed within the nuclear perimeters are considered as positive signal.
6. Repeat this process by selecting the third icon in the tab and encircling subsequent nuclei until the dataset is completed.
7. To export this data, right click on the table, select save table and save to an appropriate location.
8. To save the colocalization image, select the File menu and select 'Export…' then choose; Tagged Image File (Format) and 'Contents of Image Window – single plane (Data)' followed by clicking 'Select file name and save…', choose an appropriate location and click 'Save'.
9. Plot colocalization data in a spreadsheet followed by statistical analysis.

## Representative Results

To determine the spatial distribution of 5hmC and 5caC in differentiating hepatic progenitors immunostained slides for these DNA modifications were imaged using a microscope.

Initial analysis of the spatial distribution of these oxi-mCs was carried out through the generation of 2.5D intensity plots (Figure 1A**, 1B**). The red and green peaks appeared to be well defined with the limited presence of orange peaks indicating only a small overlap of the 5caC and 5hmC signals. In agreement with these results, the profiles for 5hmC and 5caC intensities in the corresponding cell do not strongly coincide with each other (Figure 1C). This illustrates that 5caC and 5hmC exhibit distinct patterns of nuclear distribution in hepatic progenitors suggesting that Tet-dependent oxidation of 5mC leads to generation of different oxidized derivatives of this DNA modification in specific chromatin regions.

To compare the levels of 5caC and 5hmC in Daoy medulloblastoma and BXD-1425EPN ependymoma cells, immunostaining for these oxi-mCs was carried out on both samples under identical experimental conditions. Intensities of 5caC and 5hmC signals were then compared in 20 profiles generated across 3-5 cells recorded for each cell line (Figure 2A**-2D**). The quantification of these results revealed that BXD-1425EPN cells exhibit significantly lower levels of both 5hmC and 5caC immunostaining compared to Daoy cells (Figure 2D).

Considering the two channels red (5hmC) and green (5caC) which can be denoted as R and G respectively, the Pearson's correlation coefficient (PCC) describes the degree of spatial overlap and co-segregation of R & G assuming both marks exist in a linear relationship with each other, denoted by R statistic^34^. Squaring the PCC value, denoted by R^2^ enables the measurement of green signal intensity variation which is influenced by red signal intensity^34^. This is superfluous for our 5caC vs 5hmC co-localization analysis.

To examine the spatial distribution of 5caC and 5hmC in undifferentiated (Undiff) hiPSCs and hepatic endoderm 24 hours (HE24) after induction, colocalization analysis of these DNA modifications was carried out on the same confocal images, with R values for 20 nuclei analysed for each cell line (Figure 3A**-3C**).

The quantification of these results demonstrated that degree of colocalization is significantly higher (P < 0.05) in HE24 compared to Undiff (Figure 3c). This may be attributable to the reduced magnitude of 5caC presence and thus its reduced signal intensity in Undiff cells.

**Figure:**
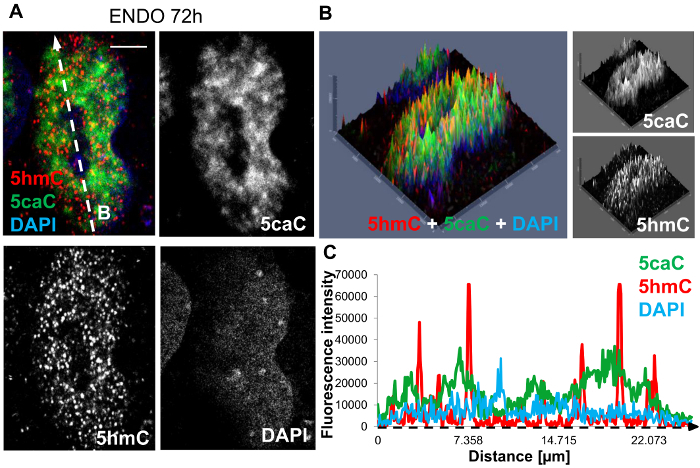

**Figure 1: Immunohistochemical staining of hepatic endoderm progenitors at 72 hours post induction of differentiation. **(**A**) Co-staining of 5hmC (red), 5caC (green) and DAPI nuclear counter stain (blue). The white dashed arrow 'B' denotes the direction of profiling sampled through the nucleus, illustrated in **1C**. Scale bar represents 5 µm. Red channel: gain 800, laser 1%. Green channel: gain 750, laser 2.4%. DAPI channel: gain 750, laser 2.6%. (**B**) 2.5D intensity plot of 5hmC, 5caC and DAPI signals. Individual peaks represent absolute signal intensities of each pixel. Merged views and individual channels are shown. (**C**) The intensity profiles of 5hmC, 5caC and DAPI signals in hepatic progenitors at 72 hours post induction of differentiation. Peak intensities were recorded along the dimensions of the white dashed arrow 'B' and are indicated along the intensity profile x-axis by a black dashed arrow. Please click here to view a larger version of this figure.

**Figure:**
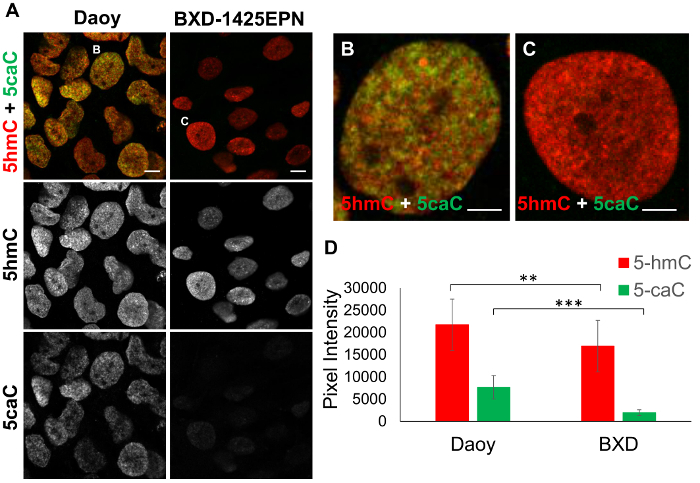

**Figure 2: Analysis of 5hmC and 5caC signal intensities in Daoy medulloblastoma and BXD-1425EPN ependymoma cell lines. **(**A**) Immunofluorescent staining of 5hmC (red) and 5caC (green) in Daoy and BXD-1425EPN cells. Images were captured with 63x oil immersion objective lens, both at the same settings. Merged views and individual channels are shown. Scale bars represent 10 µm. Red channel: gain 746, laser 1%. Green channel: gain 750, laser 2.4%. Individual (**B**) Daoy and (**C**) BXD-1425EPN cells are expanded in adjacent figures. Scale bars for (**B**) and (**C**) represent 5 µm. (**D**) Mean fluorescence intensity of 5hmC vs 5caC signals in Daoy and BXD-1425EPN cells (n = 20, SD). Significance was assessed using an F-test and Two-sample t-Test. Asterisks denote statistically significant p-values of ** as p <0.01 and *** as p <0.001. Please click here to view a larger version of this figure.

**Figure:**
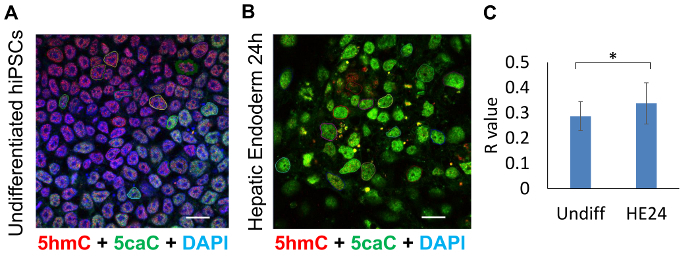

**Figure 3: Colocalization analysis of 5hmC and 5caC absolute signals in undifferentiated hiPSCs (Undiff) compared to hepatic endoderm 24 hours post-differentiated progenitor cells (HE24). **Confocal microscopy images of (**A**) undifferentiated hiPSCs and (**B**) HE24 cells stained for 5hmC (red), 5caC (green) and DAPI (blue). Cells were imaged with 63X oil immersion lens at identical settings. 20 encircled nuclei illustrate the cells sampled for colocalization analysis. Scale bars represent 20 µm. Red channel: gain 800, laser 1%. Green channel: gain 750, laser 2.4%. DAPI channel: gain 750, laser 2.6%. (**C**) Colocalization analysis of the presence of 5caC signal within range of 5hmC proximity in undiff and HE24 (n = 20, SD). Significance was assessed using an F-test and Two-sample t-Test. Asterisks denote statistically significant p-values of * p <0.05. Please click here to view a larger version of this figure.

## Discussion

This protocol describes the stepwise process of generating visual representations of DNA modifications within nuclei. Whilst sensitive and impactful, these techniques do possess a number of limitations.

It is necessary to emphasize that the data generated from 2.5D plots, signal intensity profiles and colocalization analysis are semi-quantitative in nature. Owing to the point by point illumination of the sample during image acquisition on the laser scanning confocal microscope, absolute signal intensities of each channel are recorded. However, these are not absolute magnitudes of 5hmC and 5caC being detected and only represent indications of the presence, absence or scale of these epigenetic marks.

Due to the repetitive nature of signal amplifications, limitations consistent with the magnitude of machinery applied to enhance the signal inevitably confound approximations of true physical genomic occupancy of these marks^34^. The true genomic localization of these marks may be obscured by these bulky proteins, which physically occupy areas unresolvable even at optimal confocal resolution limits of 200-300 nm^34^. Moreover, the signal intensities of the individual channels for each mark cannot be compared to each other due to differences in antibody sensitivity and fluorophore conjugation^34^. However the information obtained with imaging sheds light on the spatial and temporal organization of the genomic marks.

For accurate quantitation of 5hmC and 5caC signals, nuclei are highlighted and encircled against the DAPI counterstain, clearly demarcating the region to be analyzed. This procedure dispenses with the requirement for calibrating gating thresholds as background noise is disregarded through the manual process of selecting nuclei^1^. An automation of this process can be achieved in the latest software which allows for nuclear segmentation to be performed. Whilst free alternative software packages such as FIJI are available for colocalization analysis, drawbacks such as the requirement to convert images to binary 8 bit formats, masking images and inputting size exclusion data to select regions of interest limit the user-accessibility of this program. Additionally, considering the overall limited levels of 5hmC and 5caC in the genome coupled with their predominant location at euchromatic regions and, depletion of genomic CpG sequences in general, manual encircling of nuclei introduces user-bias. Therefore, the highlighting of nuclei automatically assumes all events occurring within the demarcated region to be positive and disregards any background pixels which may be present. Thus, analyzing images based on pixel intensity may be more meaningful. These factors are important when considering the use of Mander's colocalization coefficient to analyse the degree of spatial overlap between two signals, irrespective of their signal intensities as oppose to Pearson's correlation coefficient which assumes a linear relationship between signals.

Overall, the techniques outlined here carry the theme of visual representation of biological data in an intuitive format. Whilst semi-quantitative image analysis cannot supersede powerful techniques such as mass spectrometry in terms of sensitivity or single base resolution genome wide sequencing, it does provide complementary data allowing inferences and hypotheses to be conceived from analysis of images with precision.

## Disclosures

No potential conflicts of interest were disclosed.
